# Limited evidence for sympathetic neural overactivation in older patients with type 2 diabetes mellitus

**DOI:** 10.3389/fnins.2022.1107752

**Published:** 2023-01-12

**Authors:** Karsten Heusser, Jens Tank, André Diedrich, Annelie Fischer, Tim Heise, Jens Jordan

**Affiliations:** ^1^Institute of Aerospace Medicine, German Aerospace Center, Cologne, Germany; ^2^Vanderbilt Autonomic Dysfunction Center, Division of Clinical Pharmacology, Department of Medicine, Vanderbilt University Medical Center, Nashville, TN, United States; ^3^Department of Biomedical Engineering, School of Engineering, Vanderbilt University, Nashville, TN, United States; ^4^Profil Institut für Stoffwechselforschung GmbH, Neuss, Germany; ^5^Medical Faculty, University of Cologne, Cologne, Germany

**Keywords:** sympathetic activity, type 2 diabetes mellitus, microneurography, blood pressure, autonomic nervous system

## Abstract

**Introduction:**

Mechanistic studies suggested that excess sympathetic activity promotes arterial hypertension while worsening insulin sensitivity. Older patients with type 2 diabetes are at particularly high cardiovascular and metabolic risk. However, data on sympathetic activity in this population is scarce.

**Methods:**

We studied 61 patients with type 2 diabetes mellitus (22 women, 60.9 ± 1.4 years; 39 men, 60.9 ± 1.4 years). They had to have diabetes for at least 2 years, a hemoglobin A1c of 6.5–10%, a body-mass-index of 20–40 kg/m^2^, and had to be treated with stable doses of metformin only. We recorded ECG, finger and brachial blood pressure, and muscle sympathetic nerve activity (MSNA).

**Results:**

MSNA was 37.5 ± 2.5 bursts/min in women and 39.0 ± 2.0 bursts/min in men (*p* = 0.55). MSNA expressed as burst incidence was 52.7 ± 2.0 bursts/100 beats in women and 59.2 ± 3.1 bursts/100 beats in men (*p* = 0.21). Five out of 39 men (12.8%) and two out of 22 women (9.1%) exhibited resting MSNA measurements above the 95th percentile for sex and age. In the pooled analysis, MSNA was not significantly correlated with systolic blood pressure, diastolic blood pressure, body mass index, waist circumference, body composition, or HbA1c (*r*^2^ < 0.02, *p* > 0.26 for all).

**Discussion:**

We conclude that relatively few older patients with type 2 diabetes mellitus exhibit increased MSNA. The large interindividual variability in MSNA cannot be explained by gender, blood pressure, body mass index, or glycemic control.

## Introduction

The sympathetic nervous system could provide a mechanistic link between cardiovascular disease, particularly arterial hypertension, and type 2 diabetes mellitus. Increased sympathetic neural traffic, determined as muscle sympathetic nerve activity (MSNA), predisposes to arterial hypertension (Wallin and Sundlöf, 1979; Yamada et al., 1989), and relates to impaired insulin sensitivity (Chen et al., 2017). Conversely, increased adiposity (Grassi et al., 1995) and hyperinsulinemia (Anderson et al., 1991), which are root causes of type 2 diabetes mellitus, promote sympathetic activation. Previous smaller studies suggested that compared with healthy persons, MSNA is raised in patients with arterial hypertension or with type 2 diabetes mellitus but more so when both conditions are combined (Huggett et al., 2003; Kobayashi et al., 2010). MSNA was also increased in patients with type 2 diabetes compared with a body-mass-index matched control group without type 2 diabetes mellitus (Huggett et al., 2005). Moreover, sympathetic neural traffic and norepinephrine spillover were both increased in patients with type 2 diabetes mellitus compared to persons with impaired glucose tolerance suggesting that sympathetic activity increases with disease progression (Straznicky et al., 2012). A recent meta-analysis concluded that MSNA is increased in patients with type 2 diabetes mellitus but not in patients with type 1 diabetes mellitus (Grassi et al., 2020). Development of interventional therapies such as renal nerve ablation or electrical carotid sinus stimulation (Heusser et al., 2010) renewed interest in targeting the sympathetic nervous system in patients with cardiometabolic disease. Yet, there is large inter-individual variability in sympathetic activity in healthy persons (Keir et al., 2020). Therefore, we assessed sympathetic activity using microneurography in a larger group of patients with type 2 diabetes to test the hypothesis that sympathetic activity is increased in a substantial proportion of patients. Moreover, we tried to identify determinants of sympathetic activity in this population.

## Materials and methods

### Patients

We studied patients with type 2 diabetes on stable metformin monotherapy who had been submitted to baseline measurements for interventional studies (clinicaltrials.gov registration NCT01276288 and NCT03254849). Patients had to have hemoglobin A1c values between 6.5 and 10.0% and a BMI of 20–40 kg/m^2^. Key exclusion criteria comprised uncontrolled arterial hypertension with systolic blood pressure ≥160 mm Hg, heart failure NYHA II–IV, estimated glomerular filtration rate <60 ml/min/1.73 m^2^, myocardial infarction, stroke, or vascular interventions in the previous 12 months, or significant liver disease. Patients with a history or findings during clinical examination consistent with diabetic neuropathy were excluded. We also excluded pregnant or lactating women. The study was conducted in accordance with the Declaration of Helsinki. We obtained written informed consent from all participants before screening. The study protocol was approved by the North Rhine Medical Association (Ärztekammer Nordrhein).

### Cardiovascular autonomic testing

We studied patients while supine in the morning hours at a room temperature between 21 and 24°C. In all patients, diuretics had been discontinued. We continuously recorded respiration, ECG, thoracic impedance (Cardioscreen, Medis GmbH), and beat-by-beat finger blood pressure (Finometer, FMS). We also measured brachial blood pressure with an automated oscillometric device (Dinamap, GE Medical Systems). We recorded postganglionic, multiunit muscle sympathetic nerve activity (MSNA) from the peroneal nerve as described previously (Heusser et al., 2016; Heusser et al., 2020). Data were analog-to-digital converted and analyzed as described previously. After 20 min of rest, we obtained baseline recordings for 5 min.

### Glycemic control and body composition

We obtained fasted venous blood samples for glucose and hemoglobin A1c measurements. We measured body height with a medical gauge with shoes off and body weight on a calibrated scale with the patient in underwear. We also determined waist circumference and body composition (fat mass and lean body mass) with air displacement plethysmography (Bodpod, Cosmed).

### Statistical analysis

Descriptive statistics (mean values and SEM) were calculated for each parameter. Pearson’s correlation coefficients and corresponding *p*-values were calculated to assess the relationship between the assessments. Statistical comparisons between subgroups (men/women) were done with unpaired Student’s *t*-tests.

## Results

We included 61 patients (22 women, 60.9 ± 1.4 years; 39 men, 60.9 ± 1.4 years) with type 2 diabetes mellitus and good quality MSNA recordings in our study. Anthropometric data, resting blood pressure, heart rate, and hemoglobin A1c measurements in women, in men, and in both groups combined are provided in Table 1. At inclusion, 24 patients were on antihypertensive medications. Of those, ten patients were on two antihypertensives and three patients were on three antihypertensives (angiotensin receptor blocker = 9, angiotensin converting enzyme inhibitor = 14, beta-adrenoreceptor blocker = 5, calcium channel blocker = 8, diuretic = 1). Eight patients were on statins. Except for one woman, all were postmenopausal. Diabetes duration was on average 10.5 ± 0.7 (range 1.3–23.9) years. Data on waist circumference, fat mass, and lean body mass was available in 41 patients (16 women, 25 men). Age, body mass index, waist circumference, hemoglobin A1c, and blood pressure did not differ between women and men. Fat mass was higher and lean body mass was lower in women compared with men. Women also showed an increased heart rate compared with men.

**TABLE 1:** Patients’ characteristics.

	Males			Females			
	**Mean**	**SEM**	**Range**	**Mean**	**SEM**	**Range**	***P**-* **value**
Age (years)	60.9	1.5	41–74	60.9	1.4	43–72	0.996
BMI (kg/m^2^)	29.3	0.6	22.4–38.6	30.4	1.0	20.8–38.4	0.369
LBM (kg)	60.6	1.7	45.1–83.3	44.9	1.3	35.3–52.6	0.000
Fat mass (kg)	34.3	2.0	18.2–55.6	42.1	2.3	30.2–61.5	0.016
HbA1c (%)	7.8	0.1	6.7–9.7	8.0	0.2	6.8–9.4	0.441
SBP (mm Hg)	128.8	2.3	106.8–167.8	127.9	3.5	98.6–156.0	0.806
MAP (mm Hg)	91.8	1.4	73.8–118.4	90.1	2.2	72.2–110.7	0.506
DBP (mm Hg)	77.5	1.1	62.4–98.4	75.7	1.8	62.6–92.2	0.393
HR (beats/min)	66.9	1.5	46.8–89.1	72.3	2.3	53.6–94.5	0.044

BMI, body mass index; LBM, lean body mass; HbA1, glycated hemoglobin; SBP/MAP/DBP, systolic/mean/diastolic blood pressure; HR, heart rate.

**FIGURE 1:**
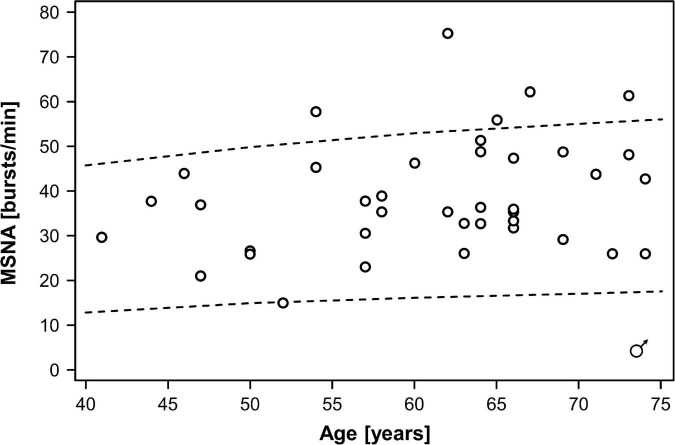
Muscle sympathetic nerve activity (MSNA) over age in men with type 2 diabetes mellitus. Dots indicate individual patients. The dashed lines indicate the 5th and the 95th percentile of MSNA in different age groups from a previous study in healthy persons (Keir et al., 2020).

**FIGURE 2:**
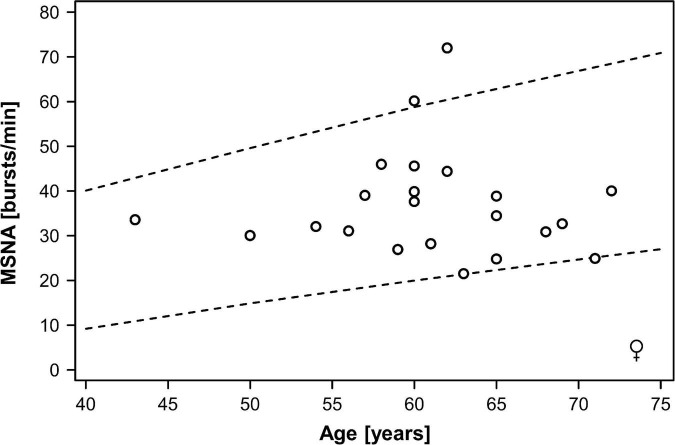
Muscle sympathetic nerve activity (MSNA) over age in women with type 2 diabetes mellitus. Dots indicate individual patients. The dashed lines indicate the 5th and the 95th percentile of MSNA in different age groups from a previous study in healthy persons (Keir et al., 2020).

In the combined dataset, MSNA ranged between 15 and 75 bursts/min, MSNA burst incidence between 26 and 98 bursts/100 beats, and MSNA burst area between 0.44 and 9.5 arbitrary units. Mean MSNA was 37.5 ± 2.5 bursts/min in women and 39.0 ± 2.0 bursts/min in men (*p* = 0.55). MSNA expressed as burst incidence was 52.7 ± 2.0 bursts/100 beats in women and 59.2 ± 3.1 bursts/100 beats in men (*p* = 0.21). The area under MSNA bursts was 2.9 ± 0.5 arbitrary units in women and 2.4 ± 0.3 arbitrary units in men (*p* = 0.35). Figures 1, 2 illustrate MSNA over age in women and in men and indicates the 5th and the 95th percentile of MSNA in different age groups from a previous study in healthy persons (Keir et al., 2020). In this previous study, age and body mass index in the older cohort (means: 62 years and 28 kg/m^2^ in women, 61 years and 27 kg/m^2^ in men) showed large overlap with our study population. Five out of 39 men (12.8%) and two out of 22 women (9.1%) exhibited resting MSNA measurements above the 95th percentile for sex and age. Overall 11.4% of all patients with type 2 diabetes had an MSNA above the 95th percentile for sex and age.

**FIGURE 3:**
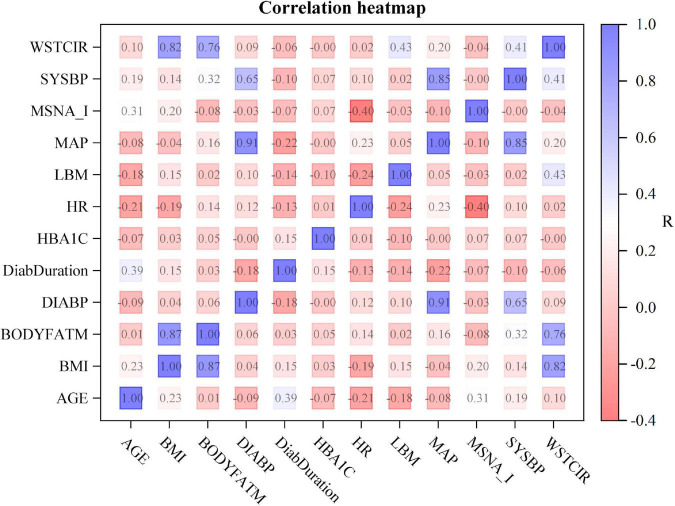
Correlation heat map showing the relationship between clinical characteristics and muscle sympathetic nerve activity expressed as burst incidence (MSNA_I). The numbers denote uncorrected *p*-values for correlations between measurements. *R*-values are color coded as shown in the scale on the right. WSTCIR, waist circumference; SYSBP, systolic blood pressure; MAP, mean arterial pressure; LBM, lean body mass; HR, heart rate; HBA1C, hemoglobin A1C; DiabDuration, duration of diabetes mellitus; DIABP, diastolic blood pressure; BODYFATM, body fat mass; BMI, body mass index.

In the pooled dataset, MSNA expressed as bursts/min was not correlated with body mass index, waist circumference, body fat, hemoglobin A1c, or diabetes duration (*r*^2^ 0.02 for all, *p* > 0.26 for all). Furthermore, MSNA expressed as bursts/min did not predict systolic blood pressure (*r*^2^ 0.0, *p* = 0.99) or diastolic blood pressure (*r*^2^ 0.0003, *p* = 0.89). Correlation analyses for MSNA bursts incidence (Figure 3) and MSNA burst area and separate analyses in women and in men yielded similar results. In addition, we divided patients into a group with lower MSNA (29 ± 1.7 bursts/min, 12 women/18 men) and a group with higher MSNA (47 ± 1.9 bursts/min, 10 women/21 men). Age was 60 ± 1.6 years in the group with lower and 61.7 ± 1.4 years in the group with higher MSNA (*p* = 0.41); body mass index was 28.9 ± 0.9 kg/m^2^ in the group with lower and 30.5 ± 0.6 kg/m^2^ in the group with higher MSNA (*p* = 0.13); blood pressure was 127 ± 2.8/76 ± 1.2 mmHg in the group with lower and 130 ± 2.6/78 ± 1.5 mmHg in the group with higher MSNA (*p* = 0.34/0.23). Similarly, there were no significant differences in waist circumference, body fat, or HbA1c between groups.

## Discussion

The important finding of our study is that in most older patients with type 2 diabetes mellitus resting MSNA is within the reference range for age and gender. Only few patients exhibit resting MSNA measurements above this reference range. Moreover, the large interindividual variability in MSNA cannot be explained by gender, blood pressure, body mass index, or glycemic control suggesting that there must be hitherto unrecognized variables affecting sympathetic activity in this population.

The sympathetic nervous system has a crucial role in cardiovascular and metabolic regulation and can contribute to cardiovascular disease progression and poor clinical outcomes. Yet, data on MSNA in patients with type 2 diabetes mellitus is surprisingly limited. For example, a recent meta-analysis on MSNA only included 35 patients with type 2 diabetes overall and 36 control persons from two studies (Grassi et al., 2020). Our study comprised 61 patients with type 2 diabetes mellitus, which is a particular strength. Our findings suggest that the degree of sympathetic activation directed to resistance vessels in type 2 diabetes is less pronounced and more heterogenous than previously believed. The finding recapitulates previous analyses on clustering of metabolic traits and blood pressure in persons with and without type 2 diabetes mellitus (Meigs et al., 1997; Gray et al., 1998; Leyva et al., 1998). These analyses suggested that metabolic traits like impaired glucose metabolism and dyslipidemia and elevated blood pressure, while having shared correlations with adiposity and hyperinsulinemia, must have more than one underlying physiological mechanism.

Impaired glucose metabolism *per se* is unlikely to drive sympathetic activation. In fact, MSNA is not increased in patients with type 1 diabetes mellitus (Grassi et al., 2020) and we did not observe a relationship between glycemic control and MSNA in our study. However, excess adiposity could increase sympathetic activity through the leptin-melanocortin system (Haynes et al., 1997). Obesity is associated with increased MSNA even before the onset of obesity-associated arterial hypertension in some (Grassi et al., 1995) but not in all studies (Narkiewicz et al., 1998). Moreover, weight loss through sleeve-gastrectomy lowers MSNA in patients with obesity (Seravalle et al., 2014). Yet, the relationship between adiposity and MSNA is strongly affected by genetic factors. While MSNA showed a positive correlation with body fat in white study participants, no such relationship was observed in Pima Indians (Weyer et al., 2000). Moreover, obesity is negatively correlated with MSNA in persons heterozygous for functional melanocortin-4 receptor mutations (Sayk et al., 2010). We speculate that the lack of significant correlations between adiposity measurements and MSNA in our study may be explained by genetic heterogeneity. An alternative explanation is that the relationship between adiposity and MSNA changes with advancing age or with progression from overweight or obesity to type 2 diabetes mellitus. Notably, body mass index explained only a small portion of MSNA variability in healthy women and none in healthy men in a large MSNA study (Keir et al., 2020).

Insulin infusion elicits peripheral vasodilation, which acutely increases MSNA through baroreflex mechanisms (Anderson et al., 1991; Haynes et al., 1997). The ability of the baroreflex to regulate MSNA appears to be maintained in patients with type 2 diabetes mellitus compared with control persons with similar body weight but without diabetes mellitus (Narkiewicz et al., 1998). While we did not measure insulin levels in our study, the mechanism could have contributed to variability in MSNA. Sympathetic excitation following glucose-mediated insulin release or during insulin infusion appears to be attenuated in insulin resistant states (Spraul et al., 1994; Vollenweider et al., 1994), which could also contribute to variability in MSNA in our patients.

Sympathetic activation raises blood pressure, however, correlations between MSNA and blood pressure across populations are generally weak (Keir et al., 2020). Thus, absence of significant correlations between adiposity measures and blood pressure in our study is not unexpected. The finding highlights the fact that the sympathetic nervous system is only one of the mechanisms regulating blood pressure and that sympathetic contributions to blood pressure exhibit large interindividual variability (Jordan et al., 2002).

An important limitation of our study is that we only measured sympathetic neural traffic directed to a relatively small vascular bed. Moreover, we cannot exclude that our results are affected by diabetic neuropathic changes in the peroneal nerve. We only assessed MSNA at rest and cannot exclude that the response to physiological stimuli may be perturbed. In fact, patients with type 2 diabetes may exhibit augmented sympathetic responses to physical exercise (Huggett et al., 2003) with diminished functional sympatholysis in the working musculature (Grotle et al., 2021). Furthermore, we only analyzed integrated MSNA signals. Single fiber MSNA recordings or more detailed MSNA raw signal analyses may contain additional information (Huggett et al., 2003; Tank et al., 2015). We did not assess how sympathetic activity is transduced to changes in vascular tone, which may be altered in patients with type 2 diabetes mellitus (Young et al., 2019). Finally, we cannot exclude that metformin treatment may have confounded our results, however, a small scale study did not show differences in MSNA, systemic norepinephrine spillover, or renal norepinephrine spillover between metformin and placebo in obese, insulin resistant men (Gudbjörnsdottir et al., 1994).

Our study, which comprises the largest sample of patients with type 2 diabetes mellitus with MSNA recordings, suggests that substantial sympathetic overactivity is not a general finding in older patients with type 2 diabetes mellitus. Instead, MSNA varies profoundly between patients and only few exhibit MSNA above the reference range. An important scientific and clinical implication is that recognition of variability in sympathetic activity could provide a frame for a more individualized treatment approach for cardiovascular and metabolic disease prevention and treatment. For example, high resting MSNA is associated with increased sympathetically mediated postprandial energy expenditure (Limberg et al., 2017). At least acutely, MSNA predicted the reduction in blood pressure through pharmacological sympathetic inhibition (Jones et al., 2001). Even in a person with MSNA within the reference range, lowering sympathetic activity could have beneficial effects. However, the response is likely more pronounced in a patient with higher MSNA, which may be particularly relevant when considering invasive treatments targeting the sympathetic nervous system.

## Data availability statement

The raw data supporting the conclusions of this article will be made available by the authors, without undue reservation.

## Ethics statement

The studies involving human participants were reviewed and approved by North Rhine Medical Association. The patients/participants provided their written informed consent to participate in this study.

## Author contributions

KH and TH: study design, conduct, analysis, and manuscript writing. JT: study design and manuscript writing. AD: analysis and manuscript writing. AF and JJ: study design, analysis, and manuscript writing. All authors contributed to the article and approved the submitted version.
